# Effect of Early Weaning on the Intestinal Microbiota and Expression of Genes Related to Barrier Function in Lambs

**DOI:** 10.3389/fmicb.2018.01431

**Published:** 2018-07-02

**Authors:** Chong Li, Weimin Wang, Ting Liu, Qian Zhang, Guoxiu Wang, Fadi Li, Fei Li, Xiangpeng Yue, Tingfu Li

**Affiliations:** ^1^The State Key Laboratory of Grassland Agro-ecosystems, Key Laboratory of Grassland Livestock Industry Innovation, Ministry of Agriculture and Rural Affairs, College of Pastoral Agriculture Science and Technology, Lanzhou University, Lanzhou, China; ^2^College of Animal Science and Technology, Gansu Agricultural University, Lanzhou, China; ^3^Engineering Laboratory of Sheep Breeding and Reproduction Biotechnology in Gansu Province, Minqin, China; ^4^Minqin Zhongtian Sheep Industry, Co., Ltd., Minqin, China

**Keywords:** gut microbiota, lamb, weaning, intestinal barrier, Toll-like receptors

## Abstract

Weaning stress has been reported to impair intestinal health. The gut microbiota plays a vital role in the long-term health of the host. However, our understanding of weaning stress on gut microbiota and barrier function is very limited in livestock species, especially lambs. We investigated the effects of early weaning stress on intestinal bacterial communities and intestinal barrier function in lambs. A total of 24 neonatal male Hu lambs were randomly allocated into two groups, one weaned on day 28 and the other weaned on day 56. At 42 and 84 days, six lambs from each group were randomly selected and sacrificed. Ileal tissue and ileal digesta were collected to compare the differences in ileal microbiota and the mRNA levels of Toll-like receptors (TLRs) and tight junction proteins between the early weaning group and the control group at day 42 when the early weaning group have been weaned for 14 days, and at day 84 when the 28 and 56 days weaning groups had been weaned for 56 and 28 days, respectively. 16S rRNA gene sequencing of ileal samples revealed that the ileal microbiota was very different between the two groups, even at 84 days of age. Early weaning significantly increased alpha diversity and altered the relative abundance of several bacterial taxa. The expression of genes related to intestinal barrier function was affected by early weaning. Early weaning significantly increased ileal mRNA levels of *TLR1* on days 42 and 84; *TLR2, TLR4*, and *TLR5* on day 84; *claudin1* and *claudin4* on day 42; and *occludin* on day 84. We demonstrate that early weaning not only altered the ileal microbiota on day 42 (compared with lambs that were not weaned), but also had lasting effects on the ileal microbiota at day 84; furthermore, early weaning impacts expression levels of genes related to intestinal barrier function.

## Introduction

Early weaning can shorten the breeding cycle of ewes in the commercial lamb industry. Thus, transitioning lambs from milk to solid feed as early as possible has traditionally been thought to increase profits. However, early weaning may be an important stressor for lambs (Orgeur et al., 1999). This stress can come as a result of lack of maternal care, discontinuation of mother–young contact, and the reluctance to eat new foods, which can lead to declining health, changes in hormone levels, and poor growth rates (Schichowski et al., 2010; Knights et al., 2012; Fazio et al., 2014; Ekiz et al., 2016). Therefore, reducing weaning stress is critical for employing a successful early weaning strategy.

Studies on early weaning in ruminants have mainly focused on growth performance, blood physiological parameters, and rumen development (Carroll et al., 2009; Schichowski et al., 2010; Knights et al., 2012; O’Loughlin et al., 2014; Lambertz et al., 2015; Ekiz et al., 2016; Liu et al., 2016); meanwhile, relatively little is known about the effect of early weaning on the intestinal barrier of ruminants. However, research does suggest that weaning stress has a remarkable effect on the intestinal barrier function of non-ruminants. Weaning stress can cause morphological and histological changes, including a reduction in villus height, an increase in crypt depth, an increase in intestinal mucosal permeability, and a variation in the mRNA levels of cytokines and tight junction proteins in the small intestine of piglets (Wijtten et al., 2011a,b; Hu et al., 2013; McLamb et al., 2013). Research in dairy calves also indicates that weaning is associated with increased gut permeability in ruminants (Wood et al., 2015). RNA-seq experiments have shown that several important pathways related to immune and inflammatory responses, especially apoptosis and pattern recognition receptor (PRR) signaling, are activated as a result of weaning and can induce small intestine atrophy in piglets (Bomba et al., 2014). PRR are a class of proteins that play a key role in the innate immune system. Toll-like receptors (TLRs) were the first family of membrane-bound PRR to be discovered; TLRs recognize structurally conserved molecules derived from microbes. Once microbes have breached the mucosal barriers of the intestinal tract, they are recognized by TLRs, which in turn activate immune cell responses. Post-weaning diarrhea (PWD) is a common problem in lambs, and the effect of early weaning on intestinal barrier function should not be ignored.

The intestinal microbiota is a complex community of microorganisms that live in the intestinal tract; these microbes play an important role in barrier function and provide many benefits to the host, including defending against pathogens, making use of all available nutrients, and secreting compounds that kill or inhibit unwelcome organisms that would compete for nutrients (Yoon et al., 2014). Disturbances to the microbiota can lead to intestinal distress. Many studies have shown that weaning can influence the intestinal microbiota of young animals (Farshim et al., 2016; Hu et al., 2016; Meale et al., 2016; Sovran et al., 2016; Chen et al., 2017; Mao et al., 2017). The intestinal microbiota is closely linked to the physiological state of the host, as well as its immune function (Turnbaugh et al., 2007; Maynard et al., 2012). Sovran et al. (2016) found that commensal intestinal species were positively correlated with early weaning stress in mice and demonstrated that the abundance of *Bacteroides* pathobionts increased before histological signs of pathology; this suggests that they may play a role in triggering the development of colitis (Sovran et al., 2016). It is important to note that weaning age may influence the intestinal microbiota and have an inflammatory effect. Furthermore, delaying weaning may decrease the inflammatory response; however, few studies have evaluated the effect of weaning on the intestinal microbiota and gut health in lambs. Understanding the impact of early weaning on the gut microbiota, as well as the role of the mucosal microbiota in host barrier function in young ruminants, is critical for ensuring health and performance throughout adulthood. We hypothesized that early weaning stress impacts intestinal barrier function, and that these alterations can modulate the bacterial community via host–microbe interactions in lambs. Therefore, the objectives of this study were to evaluate the effects of early weaning on the intestinal microbial diversity and the expression of genes related to intestinal barrier function.

## Materials and Methods

### Ethics Statement

The animal procedures used in this study were reviewed and approved by the Gansu Agricultural University’s Academic Committee and the National Natural Science Foundation of China according to guidelines established by the Biological Studies Animal Care and Use Committee of Gansu Province (Approval No. 31660670).

### Animal Handling

A total of 24 healthy neonatal male Hu lambs were selected from a commercial sheep farm (Jinchang Zhongtian Sheep Industry, Co., Ltd., Gansu, China). The animals were randomly allocated to one of two weaning regimes. In the control group (CON), lambs were weaned at 56 days-of-age (birth weight = 3.44 ± 0.44 kg); meanwhile, in the early weaning group (EW), lambs were weaned at 28 days-of-age (birth weight = 3.36 ± 0.15 kg). The lambs were housed with their ewes prior to weaning, fed a starter diet beginning at 7 days-of-age, and had *ad libitum* access to water and feed. From 59 to 69 days-of-age, the starter diet was replaced with a growing diet. Lambs were separated from their ewes and abruptly weaned at the specified time points. The formulation and chemical composition of the starter and growing diets are presented in Supplementary Table S1. The diets were formulated to meet the requirements of the “Feeding standard of meat-producing sheep and goats (NYT816-2004)” in China. Both starter and growing diets were pelletized. Ewes were fed a total mixed ration consisting of 40% corn stover silage, 12% oat grass, 10% alfalfa hay, 8% barley straw, 5% cole stalk, 13% soybean residue, 9% corn, and 3% soybean meal. Body weight and pellet feed intake were analyzed every 14 days, and have been previously reported (**Table 1**; Ma et al., 2015b).

**Table 1:** Growth performance and diet intake of lambs weaned on different days-of-age (Ma et al., 2015b).

Item	Days-of-age	CON	EW	SEM	*P*-value
Body weight (kg)	42	10.99	11.19	0.292	0.770
	84	21.55	21.63	0.595	0.964
Average daily gain (g/d)	28–42	198.57	223.57	18.442	0.483
	70–84	271.43	264.29	12.693	0.724
Average daily intake (dry matter, g/d)^∗^	28–42	152.59	205.05	-	–
	70–84	1156.41	1008.33	-	–

*The CON group was weaned on day 56; the EW group was weaned on day 28. ^∗^As the lambs were group feeding, variance analysis of average daily intake was not performed.*

### Tissue Sampling

At day 42 and day 84, six lambs from each group were randomly selected and sacrificed. The ileal digesta was collected in sterile tubes and stored at -80°C for DNA extraction to compare the differences in ileal microbiota between the early weaning group and the control group at day 42 when the early weaning group have been weaned for 14 days, and at day 84 when the 28 and 56 days weaning groups had been weaned for 56 and 28 days, respectively. The middle sections of the ileum were excised and stored at -80°C for RNA extraction to determine mRNA levels of TLRs and tight junction proteins.

### DNA Extraction and High-Throughput Sequencing

Total DNA was extracted using the Omega E.Z.N.A.^TM^ Stoll DNA kit (Omega Bio-Tek, United States). DNA quality and quantity were assessed using a NanoDrop 2000 spectrophotometer (NanoDrop Technologies, Inc., United States). Total ruminal DNA was diluted to 50 ng/L and used to prepare amplicons for high-throughput sequencing. Conventional PCR was used to amplify the V3–V4 regions of the 16S rRNA genes using the universal primers B341F (5′-TCGTCGGCAGCGTCAGATGTGTATAAGAGACAGCCTACGGGNGGCWGCAG-3′) and B785F (5′-GTCTCGTGGGCTCGGAGATGTGTATAAGAGACAGGACTACHVGGGTATCTAATCC-3′). The PCR reaction mixture (25 μL) consisted of 2 μL of DNA template, 5 μL of Q5 buffer, 5 μL of GC enhancer, 2 μL of 2.5 mM deoxynucleoside triphosphates (dNTP), 0.25 μL of Q5 DNA polymerase, and 1 μL of each primer (10 μM). Reaction conditions consisted of an initial step at 98°C for 3 min, followed by 21 cycles of 95°C for 30 s, 55°C for 45 s, and 72°C for 1 min, and then a final extension at 72°C for 7 min. Bar-coded amplicons were mixed at equal molar ratios and used for Illumina paired-end library preparation and cluster generation, and were sequenced on an Illumina MiSeq system. The sequencing data were deposited into the Sequence Read Archive (SRA) of NCBI (Accession No. SRX3366851). Raw sequences were filtered through a quality control pipeline using the Quantitative Insight into Microbial Ecology (QIIME) tool kit (Caporaso et al., 2010) and mothur (Schloss et al., 2009). Bases with quality scores > 30 were retained for further analysis. The high-quality reads were assigned to operational taxonomic units (OTUs) at a 97% identity threshold using the QIIME Uclust algorithm, and taxonomy was assigned using the RDP Bayesian classifier in mothur with a 0.80 confidence threshold. Taxonomic identification and comparisons were performed at the phylum and genus levels. Alpha diversity values were obtained using multiple diversity indices [observed species, Chao1 estimate, abundance-based coverage estimator (ACE), Shannon, and Simpson indices].

### Quantification of Total Bacteria and Select Bacterial Species

Absolute quantitative real-time PCR (qPCR) was performed to determine the 16S copy number of total bacterial species as well as the copy number of *Lactobacillus* spp., *Bifidobacterium* spp., and the *Escherichia* subgroup. The primers were previously validated (Supplementary Table S2), and qPCR was performed using a CFX96 Real-time System (Bio-Rad Laboratories, Inc., Hercules, CA, United States). The PCR conditions were: one cycle at 95°C for 3 min, 40 cycles at 95°C for 10 s, 60°C for 20 s, and 72°C for 10 s, and one cycle at 72°C for 5 min. We verified the efficiency of the primers and calculated the absolute abundances of bacteria based on standard curves. Standard curves were prepared using plasmid DNA (TransGen Biotech, Beijing, China) containing each unique 16S rDNA insert. The absolute abundances are expressed as log_10_ of copy number per gram of ileal digesta.

### mRNA Level Analysis

Total RNA was extracted using Trizol reagent (TransGen) and reverse-transcribed using the Transcript First-Strand cDNA Synthesis SuperMix Kit (TransGen). mRNA levels were quantified by real-time PCR. Primers were designed using Primer 5.0 software (PREMIER Biosoft International, Palo Alto, CA, United States) (Supplementary Table S3). Real-time PCR was performed in a CFX96 Real-time System. The PCR conditions consisted of one cycle at 95°C for 3 min, 40 cycles at 95°C for 10 s, 60°C for 20 s, and 72°C for 10 s, and one cycle at 72°C for 5 min. We verified the specificity of primers using melting curves and the fragment size of the amplification products, and verified the efficiency using standard curves prepared using plasmid DNA (TransGen) containing each gene sequence insert. We compared the stabilities and efficiencies of four candidate housekeeping genes (*GAPDH, PGK1, 18S rRNA*, and *β-actin*) in the lamb intestine and selected *β-actin* as the internal control (Ma et al., 2015a). The 2^-ΔΔCT^ method was used to analyze the data (Livak and Schmittgen, 2001).

### Statistical Analysis

Data were analyzed using the SPSS software (version 19.0; Chicago, IL, United States). The effects of weaning regimes and age on bacterial abundance were evaluated using a 2 × 2 (two weaning regimes and two animal ages) multifactorial design and false-discovery rate (FDR) was used to correct for variations in analysis (*Q* value denoted as *Q*_FDR_). The effects of weaning regimes and age on mRNA levels were evaluated using a 2 × 2 (two weaning regimes and two animal ages) multifactorial design. The data were analyzed by analysis of variance (ANOVA) using the general linear model (GLM) procedure. Statistical significance was set at *P* < 0.05.

## Results

### Alpha Diversity Measures

**FIGURE 1:**
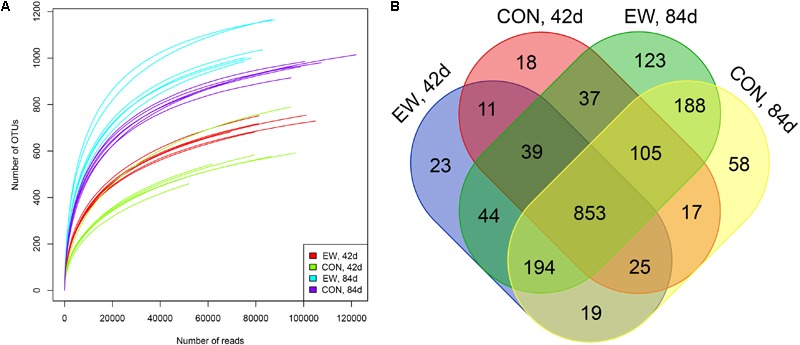
Number of operational taxonomic units (OTUs) in each group. **(A)** Rarefaction curves of OTUs; **(B)** Venn diagram of shared OTUs. EW, 42d: lambs were weaned at day 28 and sampled at day 42; EW, 84d: lambs were weaned at day 28 and sampled at day 84; CON, 42d: lambs were weaned at day 56 and sampled at day 42; CON, 84d: lambs were weaned at day 56 and sampled at day 84.

We used 16S rRNA gene sequencing of ileal digesta samples to compare the differences in ileal microbiota between the two groups at day 42 (when the early weaning group have had been weaned for 14 days but the control group was not weaned), and at day 84 (when both groups had been weaned for a relatively long time). After data filtering, quality control, and removal of low-confidence singletons, an average of 91,625 V3–V4 16S rRNA gene sequence reads was obtained for each sample. The length of the sequences ranged between 400 and 439 bp. Rarefaction curves (**Figure 1A**) revealed that there was sufficient OTU coverage to accurately describe the bacterial composition of each group. The overall number of OTUs was 1,754, and 853 shared OTUs could be detected in all groups (**Figure 1B**). The ileal microbiota was more diverse and had greater evenness in early weaned lambs compared to the control group based on the Chao1 (*P* = 0.011), ACE (*P* = 0.007), and Shannon (*P* = 0.005) indices (**Table 2**). The Chao1, ACE, Shannon, and Simpson indices all significantly increased with age (*P* < 0.001).

**Table 2:** Alpha diversity measures of bacterial communities.

Item	Day 42		Day 84			*P*-value		
	CON	EW	CON	EW	SEM	Weaning time	Age	Weaning time × Age
Coverage	0.9976	0.9980	0.9983	0.9978	<0.001	0.627	0.130	0.180
Chao1	786.16	864.89	1115.07	1181.87	13.043	0.011	<0.001	0.822
Ace	802.08	872.75	1094.59	1117.06	12.694	0.007	<0.001	0.819
Shannon	3.17	3.45	4.05	4.26	0.039	0.005	<0.001	0.721
Simpson	0.10	0.09	0.07	0.06	0.003	0.325	<0.001	0.954

*The CON group was weaned on day 56; the EW group was weaned on day 28.*

### OTU Diversity and Similarity Analysis

**FIGURE 2:**
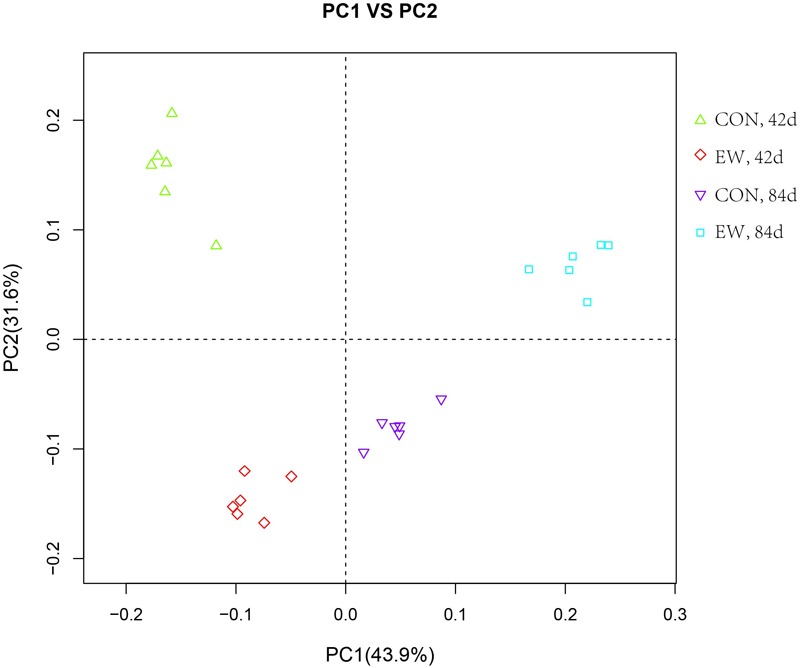
Principal coordinate analysis (PCoA) of the ileal bacterial OTUs in different groups.

Principal component analysis using the Bray–Curtis similarity method revealed that that PC1 and PC2 explained 43.9 and 31.6% of the variation between samples, respectively (**Figure 2**). As shown in the principal coordinate analysis (PCoA) figure, the plots for different groups were distinctly separated.

### Taxonomic Composition of Bacterial Populations of the Ileal Microbiota

We identified 26 phyla within the ileal microflora (**Table 3**). The relative abundances of Firmicutes, Actinobacteria, Bacteroidetes, and Proteobacteria were >1.0%. Firmicutes and Actinobacteria were the dominant phyla in all groups. Early weaning significantly increased the relative abundance of Bacteroidetes (*P* < 0.001), Proteobacteria (*P* < 0.001), Euryarchaeota (*P* = 0.001), and Cyanobacteria (*P* < 0.001), and decreased the relative abundance of Firmicutes (*P* < 0.001) and Chlamydiae (*P* < 0.001). The relative abundances of Proteobacteria (*P* < 0.001), Euryarchaeota (*P* = 0.002), Tenericutes (*P* < 0.001), Planctomycetes (*P* < 0.001), Cyanobacteria (*P* < 0.001), TM7 (*P* < 0.001), and Chlamydiae (*P* < 0.001) significantly increased with age; meanwhile, the relative abundances of Firmicutes (*P* < 0.001), Actinobacteria (*P* = 0.002), and Verrucomicrobia (*P* < 0.001) significantly decreased with age.

**Table 3:** Phylum-level taxonomic composition of the ileal bacterial communities.

Phylum	Day 42		Day 84			Weaning time		Age		W × A	
	CON	EW	CON	EW	SEM	*P*-value	Q_FDR_	*P*-value	Q_FDR_	*P*-value	Q_FDR_
*Firmicutes*	79.01	78.80	70.67	62.2	1.481	<0.001	<0.001	<0.001	<0.001	<0.001	<0.001
*Actinobacteria*	12.06	12.41	10.84	10.24	0.284	0.782	1.000	0.002	0.006	0.315	0.630
*Bacteroidetes*	1.28	4.32	2.03	5.10	0.441	<0.001	<0.001	0.242	0.485	0.979	1.000
*Proteobacteria*	0.62	1.36	2.22	4.51	0.315	<0.001	<0.001	<0.001	<0.001	<0.001	<0.001
*Euryarchaeota*	0.69	0.70	0.98	1.30	0.055	0.001	0.002	<0.001	<0.001	0.002	0.002
*Lentisphaerae*	0.47	0.55	0.53	0.56	0.036	0.180	0.721	0.807	1.000	0.373	0.745
*Verrucomicrobia*	0.52	0.33	0.08	0.25	0.042	0.781	1.000	<0.001	0.001	0.005	0.011
*Tenericutes*	0.01	0.09	0.69	0.57	0.069	0.775	1.000	<0.001	<0.001	0.156	0.313
*Planctomycetes*	0.02	0.04	0.11	0.22	0.068	0.042	0.054	<0.001	<0.001	0.046	0.551
*Cyanobacteria*	0.02	0.03	0.12	0.81	0.071	<0.001	<0.001	<0.001	<0.001	<0.001	<0.001
*TM7*	0.03	0.01	3.11	3.21	0.347	0.870	1.000	<0.001	<0.001	0.824	1.000
*Chlamydiae*	0	0	0.63	0	0.061	<0.001	<0.001	<0.001	<0.001	<0.001	<0.001
Other (<0.1%)	0.06	0.07	0.06	0.10	0.005	0.579	0.772	<0.001	<0.001	<0.001	<0.001
Unclassified	5.12	1.52	7.84	10.98	0.751	0.006	0.025	0.019	0.028	0.021	0.028

*The CON group was weaned on day 56; the EW group was weaned on day 28.*

We identified 372 genera within the ileal microflora (**Table 4**); 40 of these genera had relative abundances >0.05%. Many sequences remained unclassified. *Turicibacter* and *Olsenella* were the dominant genera across all groups. Amongst the 40 genera with relative abundances >0.05%, early weaning significantly increased the relative abundance of *Bifidobacterium* (*P* < 0.001), *Butyrivibrio* (*P* < 0.001), *Prevotella* (*P* < 0.001), *Acetobacterium* (*P* < 0.001), *Bacteroides* (*P* < 0.001), *Aeriscardovia* (*P* < 0.001), *Lactococcus* (*P* < 0.001), *Methanobrevibacter* (*P* < 0.001), *Succinivibrio* (*P* < 0.001), *Escherichia/Shigella* (*P* = 0.001), *Streptococcus* (*P* < 0.001), *Pseudoflavonifractor* (*P* < 0.001), *Succiniclasticum* (*P* = 0.001), and *Ruminococcus* (*P* < 0.001), and decreased the abundance of *Turicibacter* (*P* < 0.001), *Clostridium* (*P* < 0.001), *Sharpea* (*P* < 0.001), *Syntrophococcus* (*P* < 0.001), *Arthromitus* (*P* < 0.001), *Pseudomonas* (*P* < 0.001), *Cellulosilyticum* (*P* < 0.001), *Anaerovibrio* (*P* < 0.001), *Lactobacillus* (*P* = 0.010), *Chlamydophila* (*P* < 0.001), and *Allobaculum* (*P* < 0.001). Amongst the 40 genera with relative abundances >0.05%, the relative abundance of *Turicibacter* (*P* = 0.006), *Butyrivibrio* (*P* < 0.001), *Prevotella* (*P* = 0.001), *Aeriscardovia* (*P* < 0.001), *Lactococcus* (*P* < 0.001), *Methanobrevibacter* (*P* < 0.001), *Akkermansia* (*P* < 0.001), *Succinivibrio* (*P* < 0.001), *Arthromitus* (*P* < 0.001), *Pseudoflavonifractor* (*P* = 0.004), *Succiniclasticum* (*P* < 0.001), *Adlercreutzia* (*P* < 0.001), *Anaerovibrio* (*P* = 0.001), *Saccharimonas* (*P* < 0.001), *Chlamydophila* (*P* < 0.001), and *Mycoplasma* (*P* < 0.001) significantly increased with age, and the relative abundance of *Megasphaera* (*P* < 0.001), *Clostridium* (*P* = 0.009), *Sharpea* (*P* < 0.001), *Acetobacterium* (*P* < 0.001), *Syntrophococcus* (*P* < 0.001), *Akkermansia* (*P* < 0.001), *Escherichia/Shigella* (*P* < 0.001), *Howardella* (*P* < 0.001), *Bulleidia* (*P* < 0.001), *Streptococcus* (*P* = 0.001), *Pseudomonas* (*P* < 0.001), *Cellulosilyticum* (*P* < 0.001), *Mitsuokella* (*P* < 0.001), and *Allobaculum* (*P* < 0.001) significantly decreased with age.

**Table 4:** Genus-level taxonomic composition of the ileal bacterial communities.

Genus	Day 42		Day 84			Weaning time		Age		W × A	
	CON	EW	CON	EW	SEM	*P*-value	Q_FDR_	*P*-value	Q_FDR_	*P*-value	Q_FDR_
*Turicibacter*	10.62	12.86	21.26	7.88	1.128	<0.001	<0.001	0.006	0.008	<0.001	<0.001
*Olsenella*	4.91	4.51	4.41	3.87	0.215	0.300	0.600	0.189	0.600	0.848	1.000
*Bifidobacterium*	0.61	3.93	1.18	0.54	0.294	<0.001	<0.001	<0.001	<0.001	<0.001	<0.001
*Megasphaera*	2.00	2.12	0.05	0.20	0.22	0.478	0.957	<0.001	<0.001	0.951	1.000
*Clostridium*	3.07	1.76	2.54	0.79	0.217	<0.001	<0.001	0.009	0.019	0.400	0.533
*Butyrivibrio*	0.15	1.72	1.47	1.44	0.135	<0.001	<0.001	<0.001	<0.001	<0.001	<0.001
*Sharpea*	2.54	1.10	0.61	0.32	0.182	<0.001	<0.001	<0.001	<0.001	<0.001	<0.001
*Prevotella*	0.43	0.95	0.93	1.01	0.059	<0.001	0.002	0.001	0.002	0.007	0.009
*Acetobacterium*	0.45	0.75	0.19	0.11	0.054	<0.001	0.007	<0.001	<0.001	0.090	<0.001
*Bacteroides*	0.15	0.72	0.04	1.05	0.123	<0.001	0.002	0.568	0.757	0.258	0.516
*Aeriscardovia*	0.38	0.66	0.92	1.06	0.058	<0.001	<0.001	<0.001	<0.001	0.090	0.120
*Lactococcus*	0.22	0.58	0.83	0.65	0.048	0.010	0.013	<0.001	<0.001	<0.001	<0.001
*Syntrophococcus*	1.39	0.54	0.20	0.61	0.093	<0.001	<0.001	<0.001	<0.001	<0.001	<0.001
*Methanobrevibacter*	0.23	0.43	0.76	1.12	0.072	<0.001	<0.001	<0.001	<0.001	0.010	0.014
*Akkermansia*	0.50	0.32	0.08	0.24	0.041	0.829	1.000	<0.001	0.001	0.007	0.014
*Succinivibrio*	0.06	0.32	1.08	2.58	0.213	<0.001	<0.001	<0.001	<0.001	<0.001	<0.001
*Escherichia/Shigella*	0.19	0.28	0.01	0.16	0.025	0.001	0.002	<0.001	0.001	0.354	0.472
*Howardella*	0.22	0.16	0.05	0.09	0.018	0.885	1.000	<0.001	0.001	0.084	0.169
*Bulleidia*	0.22	0.13	0.08	0.01	0.018	<0.001	<0.001	<0.001	<0.001	0.499	0.665
*Parabacteroides*	0.01	0.11	0.01	0.17	0.026	0.023	0.051	0.543	0.736	0.552	0.736
*Streptococcus*	0.03	0.10	0.05	0.04	0.007	<0.001	<0.001	0.001	0.002	<0.001	<0.001
*Arthromitus*	0.41	0.09	2.86	0.01	0.25	<0.001	<0.001	<0.001	<0.001	<0.001	0.007
*Pseudoflavonifractor*	0.01	0.09	0.05	0.23	0.022	<0.001	0.001	0.004	0.008	0.101	0.134
*Pseudomonas*	0.18	0.08	0.08	0.02	0.013	<0.001	<0.001	<0.001	<0.001	0.140	0.187
*Succiniclasticum*	0.07	0.07	0.08	0.20	0.013	0.001	0.001	<0.001	0.001	0.001	0.001
*Cellulosilyticum*	0.24	0.07	0.04	0.01	0.02	<0.001	<0.001	<0.001	<0.001	<0.001	0.001
*Ruminococcus*	0.01	0.07	0.05	0.11	0.015	0.039	0.156	0.198	0.396	0.872	1.000
*Eggerthella*	0.07	0.06	0.07	0.05	0.003	0.035	0.070	0.013	0.063	0.228	0.303
*Adlercreutzia*	0.01	0.06	0.15	0.16	0.014	0.024	0.054	<0.001	<0.001	0.098	0.130
*Mitsuokella*	0.35	0.05	0.10	0.02	0.029	<0.001	<0.001	<0.001	<0.001	<0.001	<0.001
*RC9-group*	0.14	0.05	0.06	0.04	0.013	0.079	0.158	0.018	0.072	0.125	0.166
*Alistipes*	0.01	0.04	0.03	0.32	0.065	0.214	0.419	0.243	0.419	0.314	0.419
*Anaerovibrio*	0.05	0.04	0.10	0.06	0.007	0.024	0.054	0.001	0.003	0.115	0.153
*Lactobacillus*	2.18	0.01	0.03	0.02	0.197	<0.001	<0.001	<0.001	<0.001	<0.001	<0.001
*Saccharimonas*	0.02	0.01	3.11	3.21	0.348	0.873	1.000	<0.001	<0.001	0.821	1.000
*Chlamydophila*	0	0	0.62	0	0.06	<0.001	<0.001	<0.001	<0.001	<0.001	<0.001
*Mycoplasma*	0	0	0.25	0.3	0.038	0.632	0.847	<0.001	<0.001	0.635	0.847
*Allobaculum*	0.35	0	0	0	0.033	<0.001	<0.001	<0.001	<0.001	<0.001	<0.001
Other (<0.05%)	0.70	0.87	1.10	2.02	0.112	<0.001	<0.001	<0.001	<0.001	<0.001	<0.001
Unclassified	67.49	64.18	54.19	68.49	1.275	<0.001	<0.001	<0.001	<0.001	<0.001	<0.001

*The CON group was weaned on day 56; the EW group was weaned on day 28.*

### Quantification of Total and Selected Bacteria by qPCR

Weaning time (*P* = 0.751) and age (*P* = 0.913) had no effect on total bacterial copy number; however, there was a significant interactive effect (*P* = 0.002) (**Table 5**). The total bacterial copy number in the EW group was significantly lower than that in the control group on day 42 (*P* = 0.024), but significantly higher than that in the CON group on day 84 (*P* = 0.018). Early weaning significantly decreased the relative abundance (determined via qPCR) of *Lactobacillus* spp. (*P* = 0.001). Weaning time (*P* = 0.144) and age (*P* = 0.220) had no effect on the abundance of the *Escherichia* subgroup; however, there was a significant interactive effect (*P* = 0.023). The relative abundance of the *Escherichia* subgroup in the EW group was significantly higher than that in the CON group on day 84 (*P* = 0.013); however, there was no significant difference between the two groups on day 42 (*P* = 0.453).

**Table 5:** Absolute abundance of total bacteria (log_10_ copy number per gram of ileal digesta), as well as the relative abundance of selected bacteria within the ileal bacterial communities (%).

Taxon	Day 42		Day 84		SEM	*P*-value		
	CON	EW	CON	EW		Weaning time	Age	W × A
Total bacteria	11.37	11.05	11.07	11.34	0.041	0.751	0.913	0.002
*Lactobacillus* spp.	2.13	0.12	1.15	0.16	0.171	0.001	0.191	0.162
*Bifidobacterium* spp.	0.95	1.24	1.29	0.25	0.307	0.291	0.551	0.604
*Escherichia* subgroup	0.17	0.13	0.03	0.18	0.018	0.144	0.220	0.023

*Escherichia subgroup was composed of *E. coli, Hafnia alvei* and *Shigella* spp. The relative abundances are expressed as percentages, calculated by dividing the 16S rRNA gene copy number of each bacterium/group by the 16S rRNA gene copy number of total bacteria. The CON group was weaned on day 56; the EW group was weaned on day 28.*

### Toll-Like Receptor and Tight Junction Protein mRNA Levels

**FIGURE 3:**
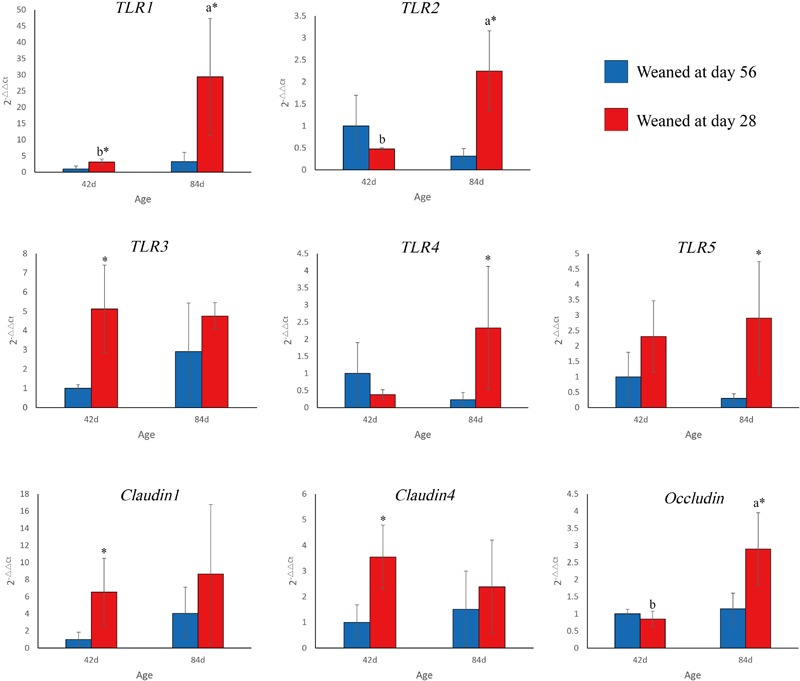
*TLR1–5* and tight junction proteins gene expression in the ileum. Error bars show standard deviation. ^a,b^Columns with different letters at different age points across the clusters of bars indicate significant differences (*P* < 0.05). ^∗^Columns with asterisks indicate significant differences within a cluster of bars (*P* < 0.05).

Weaning time and age had a substantial effect on *TLR* and tight junction protein mRNA levels in the ileum (**Figure 3**). *TLR1* mRNA levels in the EW group were higher than those in the CON group on days 42 (*P* = 0.046) and 84 (*P* = 0.025). In the EW group, *TLR1* mRNA levels were significantly higher on day 84 than on day 42 (*P* = 0.049). *TLR2* mRNA levels in the EW group were higher than those in the CON group on day 84 (*P* = 0.023). In the EW group, *TLR2* mRNA levels were significantly higher on day 84 than on day 42 (*P* = 0.018). *TLR3* mRNA levels in the EW group were higher than those in the CON group on day 42 (*P* = 0.036). *TLR4* (*P* = 0.038) and *TLR5* (*P* = 0.048) mRNA levels in the EW group were higher than those in the CON group on day 84. *Claudin1* (*P* = 0.041) and *claudin4* (*P* = 0.025) mRNA levels in the EW group were higher than those in the CON group on day 42. *Occludin* mRNA levels were higher in the EW group than in the CON group on day 84 (*P* = 0.049). In the EW group, *occludin* mRNA levels mRNA levels were significantly higher on day 84 than on day 42 (*P* = 0.018).

### Relationship Between the Bacterial Community and mRNA Levels Related to the Intestinal Barrier

**FIGURE 4:**
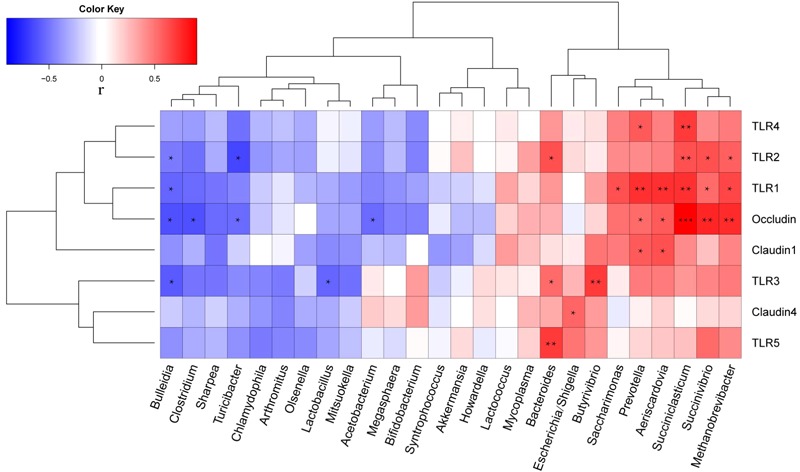
Correlation coefficients between relative abundances of ileal bacterial genera (>0.1% relative abundance) and expression of mRNA (2^-ΔΔCT^) related to intestinal barrier function. ^∗^*P* < 0.05, ^∗∗^*P* < 0.01, ^∗∗∗^*P* < 0.001.

Correlation analysis of the relative abundances of genera with relative abundance >0.1% and mRNA levels (2^-ΔΔCT^) of all lambs on days 42 and 84 was performed using Spearman’s correlation test (**Figure 4**). *TLR1* level was positively correlated with the relative abundances of *Saccharimonas* (*r* = 0.618, *P* = 0.019), *Succinivibrio* (*r* = 0.538, *P* = 0.047), *Prevotella* (*r* = 0.733, *P* = 0.003), *Aeriscardovia* (*r* = 0.720, *P* = 0.0049), *Methanobrevibacter* (*r* = 0.655, *P* = 0.011), and *Succiniclasticum* (*r* = 0.729, *P* = 0.003); it was negatively correlated with the relative abundance of *Bulleidia* (*r* = -0.551, *P* = 0.041). *TLR2* was positively correlated with the relative abundances of *Succinivibrio* (*r* = 0.616, *P* = 0.019), *Methanobrevibacter* (*r* = 0.559, *P* = 0.038), *Bacteroides* (*r* = 0.620, *P* = 0.018), and *Succiniclasticum* (*r* = 0.629, *P* = 0.016); it was negatively correlated with the relative abundance of *Turicibacter* (*r* = -0.652, *P* = 0.012). *TLR3* was positively correlated with the relative abundances of *Butyrivibrio* (*r* = 0.698, *P* = 0.006) and *Bacteroides* (*r* = 0.620, *P* = 0.018); it was negatively correlated with the relative abundances of *Lactobacillus* (*r* = -0.548, *P* = 0.043) and *Bulleidia* (*r* = -0.580, *P* = 0.030). *TLR4* was positively correlated with the relative abundances of *Prevotella* (*r* = 0.578, *P* = 0.030) and *Succiniclasticum* (*r* = 0.690, *P* = 0.006). *TLR5* was positively correlated with the relative abundance of *Bacteroides* (*r* = 0.689, *P* = 0.006). *Occludin* was positively correlated with the relative abundances of *Succinivibrio* (*r* = 0.769, *P* = 0.001), *Prevotella* (*r* = 0.535, *P* = 0.049), and *Succiniclasticum* (*r* = 0.900, *P* < 0.001); it was negatively correlated with the relative abundances of *Turicibacter* (*r* = -0.534, *P* = 0.049), *Clostridium* (*r* = -0.626, *P* = 0.017), *Aeriscardovia* (*r* = -0.593, *P* = 0.026), and *Bulleidia* (*r* = -0.628, *P* = 0.016). *Claudin1* was positively correlated with the relative abundances of *Prevotella* (*r* = 0.590, *P* = 0.026) and *Aeriscardovia* (*r* = 0.626, *P* = 0.017). Finally, *claudin4* was positively correlated with the relative abundance of *Escherichia/Shigella* (*r* = 0.551, *P* = 0.041).

## Discussion

Understanding the impact of early weaning on the gut microbiota in young ruminants, as well as the role of mucosal microbiota in host barrier function, is necessary for optimizing health and performance throughout adulthood. We characterized the effects of early weaning on the ileal microbiota composition and the expression levels of genes related to barrier function. We compared the short- and long-term effects of early weaning. At day 84, the lambs in the control and early weaning groups had the same dietary composition and similar feed intake levels.

Consistent with previous studies on the calf intestinal microbiota (Castro et al., 2016; Bi et al., 2017), Firmicutes and Actinobacteria were the dominant phyla in the ileal microbiota in all groups. *Turicibacter* was the dominant genus; this finding is not in line with other studies. *Turicibacter* is a genus in the phylum Firmicutes that has most commonly been found in the guts of animals (Auchtung et al., 2016); however, the specific metabolic properties of this genus are still unknown. In a previous study in calves, *Olsenella* was the dominant genus in jejunal digesta (Bi et al., 2017); however, in our study *Olsenella* was the second most dominant genus. Although the dominant phyla and genus was the same for both groups on days 42 and 84 in our study, each group had a distinct ileal microbiota composition by age. This was determined based on alpha diversity indices and by the clustering of samples by group in PCoA. A parallel study to this work (Meale et al., 2016) also showed that development of the fecal microbiome was affected by weaning in dairy calves. In the current study, most of the alpha diversity indices (Chao1, ACE, and Shannon indices) increased with age, which suggests that the microbiota was more diverse at 84 days than at 42 days-of-age. This is congruent with the process of microbial colonization observed in ruminal contents (Jami et al., 2013; Jiao et al., 2015) and the intestinal digesta of ruminants (Castro et al., 2016; Meale et al., 2016). In theory, a high level of diversity provides “functional redundancy” that allows an ecosystem to be resilient and stable to environmental stressors (Konopka, 2009). Some genera that are efficient carbohydrate degraders and short chain fatty acid (SCFA) producers, such as *Butyrivibrio* and *Prevotella*, significantly increased with age; this also indicates a shift toward a more adult ruminant-like intestinal environment associated with an increased capacity for carbohydrate degradation. In this study, early weaning significantly increased the Chao1, ACE, and Shannon indices; this shows that the ileal microbiota in early-weaned lambs is more diverse. A highly diverse gut microbiota is generally considered beneficial for host health and is also regarded as a sign of a mature gut microbiota (Turnbaugh et al., 2007; Le Chatelier et al., 2013). However, some studies have found that premature development and diversification of the microbiota may negatively impact immune function (Nylund et al., 2013; Wood et al., 2015). It is also possible that a highly diverse and rich bacterial community is not beneficial for the immature intestinal tract of young animals. In this study, early weaning significantly increased the relative abundance of Proteobacteria. Proteobacteria include a wide variety of pathogens, such as *Escherichia, Salmonella, Vibrio*, and *Helicobacter* (Chen et al., 2017); this indicates that early weaning might increase the abundance of opportunistic pathogens. *Lactobacillus* and *Bifidobacterium* have been linked to increased resistance to infection and diarrheal disease, as well as the stimulation of immune system activity, which are possibly due to the chemical composition and structure of their cell wall components. Some *Lactobacillus* and *Bifidobacterium* species form substances that are antagonistic toward other organisms, such as produce organic acids and bacteriocins (Macfarlane et al., 2008; Vizoso Pinto et al., 2009). In our study, early weaning significantly decreased the abundance of *Lactobacillus*. In the control group of our study, the abundance of *Bifidobacterium* significantly increased with age; however, in the early weaning group, the abundance of *Bifidobacterium* significantly decreased with age. We also found that early weaning significantly increased the abundance of *Escherichia coli*. It seems that in this study, early weaning had a negative impact on the maturation of the gut microbiota.

In this study, the difference in microbial composition between the two groups on day 42 was due to the change in feeding pattern. Mounting evidence suggests that diet impacts the intestinal microbial community (Liu et al., 2017), and weaning is a multifaceted transformation that includes the adjustment to a non-milk diet. Interestingly, the intestinal microbiota was very different on day 84, even though the groups weaned on day 28 and day 56 had the same diet structure and similar feed intakes. Thus, differences in microflora were mainly due to the influence of early microbial colonization and host physiological conditions. Considering the significant changes in the intestinal microflora between days 42 and 84, we believe that the differences in intestinal microflora are mainly due to host–microbe interactions and are influenced by the physiological state of the host. Furthermore, we believe that the most significant host–microbe interactions occur at the intestinal barrier.

Weaning typically combines a number of physical and psychological stressors that can alter the immune state and increase susceptibility to disease. Studies have shown that the systemic reaction of ruminants to weaning stress encompasses a wide range of endocrinological, immunological, and inflammatory responses (Carroll et al., 2009; O’Loughlin et al., 2014; Ranade et al., 2014; Lambertz et al., 2015). Additionally, Meale et al. (2017) showed that weaning age may influence the inflammatory response of the lower gut; however, no firm conclusions could be drawn in that regard from that experiment because this was not specifically studied. We further analyzed the effect of weaning age on the expression of genes associated with intestinal barrier function and the microbial flora. TLRs can detect a wide variety of pathogen-associated molecular patterns (PAMPs) from bacteria, viruses, fungi, and parasites. Each TLR has a distinct repertoire of specificities for conserved PAMPs; for example, TLR1 binds triacyl lipopeptides, TLR2 mainly binds peptidoglycans and lipoproteins, TLR3 binds double-stranded RNA, TLR4 mainly binds lipopolysaccharide, and TLR5 binds flagellin. Once pathogens have breached the mucosal barriers of the intestinal tract, they are recognized by TLRs, which in turn activate immune cell responses. In this study, early weaning significantly increased the ileal mRNA levels of *TLR1* on days 42 and 84; *TLR3* on day 42; and *TLR2, TLR4*, and *TLR5* on day 84. The increased expression of *TLR* genes in the early weaning group suggests that weaning stress activates innate immune responses in the intestine. Meale et al. (2017) also reported that host PRRs were stimulated post-weaning. However, another study reported that most TLRs were significantly downregulated after weaning. This study also concluded that the downregulation of TLRs in weaned calves might reflect a developmental shift toward a mature ruminant to prevent unnecessary inflammatory responses to the changing intestinal microbiome (Malmuthuge et al., 2012). In our study, most TLRs in the early weaning group were significantly upregulated compared with the control group on day 84. This coincides with a drastic change in the ileal microbiota and indicates that early weaning has a lasting effect on TLR expression. Recent studies have shown that feeding a starter diet in combination with a milk replacer near weaning leads to the upregulation of TLR genes, indicating that starter feeding stimulates the expression of host PRRs (Malmuthuge et al., 2013). Weaning stress may allow more bacterial products to breach the mucosal barrier to stimulate the expression of TLR genes. In dairy calves, it has recently been shown that weaning is associated with increased permeability of the lower gut (Wood et al., 2015). The gastrointestinal tract is lined by strong barrier layers of epithelial cells stitched together by tight junctions that prevent pathogens from squeezing between them and entering the body. Tight junction proteins, such as claudins and occludins, form the primary physical barrier and decrease the permeability of the intestinal tract (Li et al., 2012). Malmuthuge et al. (2013) found that the expression of tight junction protein coding genes was affected by the addition of calf starter to a diet of milk replacer during weaning. In our study, early weaning significantly increased the ileal mRNA levels of *claudin1* and *claudin4* on day 42, and *occludin* on day 84. This might be due to an increase in the proliferation and differentiation of ileal epithelial cells. Weaning stress could cause changes in intestinal histology, including an increase in crypt depth, which usually corresponds to an increase in the proliferation and differentiation of stem cells residing at the base of the intestinal glands (Shyer et al., 2015).

Interestingly, our study shows that the effect of early weaning on TLRs and occludin gene expression is not immediate. Rather it was most apparent on day 84, when the early weaning group and the control group had the same diet structure and similar feed intakes. These results indicate that early weaning has a lasting effect on intestinal barrier function that coincide with changes to the microbiota. It is important to note that early weaning may have a lasting and profound effect on gut health, and that the microflora may have a close relationship with intestinal barrier function. We further assessed the correlation between the relative abundances of bacterial genera and the expression of genes related to the intestinal barrier using Spearman’s correlation analysis and found that several bacterial genera were significantly correlated with gene expression levels of TLRs and tight junction proteins. Recent studies also reported that *TLR* expression was correlated with several bacterial taxa (Jiao et al., 2017; Liu et al., 2017). However, considering the vast differences in microbiome composition between the different groups, we cannot infer specific links through correlation due to large fluctuations in the microbiome between groups. This would likely yield inaccurate correlations due to strong habitat filtering (Berry and Widder, 2014) and the heteroscedasticity of the data. Although the correlation relationship does not necessarily indicate a direct causal effect, the observed multiple significant correlations between relative abundances of microbial taxa and mRNA levels of *TLRs* and tight junction proteins provide some insight into potential host–microbiotic interactions in the gut. Although there have been many studies focusing on the gut microbiota in ruminants, only a few have attempted to integrate the gut microbiota composition with the host (Malmuthuge and Guan, 2017). Although our study indicates that early weaning has lasting effects on both the ileal microbiota composition and the expression of *TLRs* and tight junction protein genes, and we inferred that the differences in intestinal microflora may be due to host–microbe interactions, integrated approaches are required to understand host–microbial interactions during weaning stress and inform weaning strategies and/or manipulate the gut microbiome to improve lamb health.

## Conclusion

Early weaning significantly increases bacterial diversity and alters the relative abundance of several dominant taxa in the ileum of lambs. Meanwhile, early weaning increased the expression of *TLRs* and tight junction protein genes. Early weaning not only altered the ileal microbiota composition on day 42 (compared with lambs that were not weaned), but also had lasting effects on the ileal microbiota composition and barrier function on day 84.

## Author Contributions

CL and FDL designed the study. CL, QZ, GW, TL, and TFL performed the experiments and collected sample. CL, FL, and XY analyzed the data. CL and WW wrote the manuscript. All authors contributed to manuscript revision, read and approved the submitted version.

## Conflict of Interest Statement

The authors declare that the research was conducted in the absence of any commercial or financial relationships that could be construed as a potential conflict of interest.
