# Supplementary material for: Effect of Early Weaning on the Intestinal Microbiota and Expression of Genes Related to Barrier Function in Lambs
Source: Front Microbiol. 2018 Jul 2;9:1431. doi: 10.3389/fmicb.2018.01431 (PMC6036172; doi:10.3389/fmicb.2018.01431)
Supplement: Supplementary file 1 [file Table_1.DOCX]

**Table S1.** Feed formulation and chemical composition of two diets (air-dry basis).

| Items | Starter | Growing diet |
| --- | --- | --- |
| Ingredients [%] |  |  |
| Alfalfa hay | 5.00 | 25.00 |
| Malt sprouts (dried) | 17.00 | 18.00 |
| Corn | 55.90 | 44.50 |
| Soybean meal | 11.00 | 9.00 |
| Whey powder | 1.50 | - |
| Extruded soybean | 7.00 | - |
| Limestone | 1.20 | 0.70 |
| NaHCO_3_ | - | 1.40 |
| Premix* | 1.00 | 1.00 |
| NaCl | 0.30 | 0.40 |
| Sweetener | 0.10 | - |
| Total | 100 | 100 |
| Chemical composition ^#^ |  |  |
| Dry matter [%] | 87.48 | 87.61 |
| Crude protein [%] | 18.51 | 14.64 |
| Digestible energy [MJ/kg] | 13.50 | 12.07 |
| Neutral detergent fibre [%] | 18.00 | 22.00 |
| Starch [%] | 18.81 | 28.93 |
| Ca [%] | 0.67 | 0.70 |
| P [%] | 0.32 | 0.35 |
| Concentrate-to-forage ratio | 78:22 | 57:43 |

Notes: *The premix is (per kilogram of diet): 25 mg Fe as FeSO_4_⋅H_2_O; 40 mg Zn as ZnSO_4_⋅H_2_O; 8 mg Cu as CuSO_4_⋅5H_2_O; 40 mg Mn as MnSO_4_⋅H_2_O; 0.3 mg I as KI; 0.2 mg Se as Na_2_SeO_3_; 0.1 mg Co as CoCl_2_; 940 IU vitamin A; 111 IU vitamin D; 20 IU vitamin E, and; 0.02 mg vitamin B_12_. ^#^ Dry matter, crude protein, neutral detergent fiber, Ca, and P were measured. Digestible energy and starch were calculated from data provide by the Feed Database of China (2013).
