# Supplementary material for: Effect of Early Weaning on the Intestinal Microbiota and Expression of Genes Related to Barrier Function in Lambs
Source: Front Microbiol. 2018 Jul 2;9:1431. doi: 10.3389/fmicb.2018.01431 (PMC6036172; doi:10.3389/fmicb.2018.01431)
Supplement: Supplementary file 2 [file Table_2.docx]

**Table S2.** Primers used for quantification of total bacteria and select bacteria.

| Target | Primer sequence | Size, bp | Amplification efficiency | Reference |
| --- | --- | --- | --- | --- |
| Total bacteria | F: 5' TCCTACGGGAGGCAGCAGT 3' | 466 | 93.18% | (Nadkarni et al., 2002) |
|  | R: 5' GGACTACCAGGGTATCTAATCCTGTT 3' |  |  |  |
| *Lactabacillus spp.* | F: 5' AGCAGTAGGGAATCTTCCA 3' | 340 | 92.09% | (Walter et al., 2001) |
|  | R: 5' CACCGCTACACATGGAG 3' |  |  |  |
| *Bifidobacterium spp.* | F: 5' GGGTGGTAATGCCGGATG 3' | 523 | 90.31% | (Kok et al., 1996) |
|  | R: 5' CCACCGTTACACCGGGAA 3' |  |  |  |
| *Escherichia* subgroup^*^ | F: 5' GTTAATACCTTTGCTCATTGA 3' | 340 | 98.80% | (Malinen et al., 2003) |
|  | R: 5' ACCAGGGTATCTAATCCTGTT 3' |  |  |  |

Note: ^*^*Escherichia* subgroup composed of *E. coli, Hafnia alvei* and *Shigella* spp.

# References

Kok, R.G., de Waal, A., Schut, F., Welling, G.W., Weenk, G., and Hellingwerf, K.J. (1996). Specific detection and analysis of a probiotic Bifidobacterium strain in infant feces. *Appl Environ Microbiol* 62(10)**,** 3668-3672.

Malinen, E., Kassinen, A., Rinttila, T., and Palva, A. (2003). Comparison of real-time PCR with SYBR Green I or 5'-nuclease assays and dot-blot hybridization with rDNA-targeted oligonucleotide probes in quantification of selected faecal bacteria. *Microbiology* 149(Pt 1)**,** 269-277. doi: 10.1099/mic.0.25975-0

Nadkarni, M.A., Martin, F.E., Jacques, N.A., and Hunter, N. (2002). Determination of bacterial load by real-time PCR using a broad-range (universal) probe and primers set. *Microbiology* 148(Pt 1)**,** 257-266. doi: 10.1099/00221287-148-1-257

Walter, J., Hertel, C., Tannock, G.W., Lis, C.M., Munro, K., and Hammes, W.P. (2001). Detection of Lactobacillus, Pediococcus, Leuconostoc, and Weissella species in human feces by using group-specific PCR primers and denaturing gradient gel electrophoresis. *Appl Environ Microbiol* 67(6)**,** 2578-2585. doi: 10.1128/AEM.67.6.2578-2585.2001
