# Supplementary material for: Effect of Early Weaning on the Intestinal Microbiota and Expression of Genes Related to Barrier Function in Lambs
Source: Front Microbiol. 2018 Jul 2;9:1431. doi: 10.3389/fmicb.2018.01431 (PMC6036172; doi:10.3389/fmicb.2018.01431)
Supplement: Supplementary file 3 [file Table_3.DOCX]

**Table S3.** Sequences of forward and reverse primers used for real-time PCR.

| Target | GeneBank number | Primer sequence | Size, bp | Amplification efficiency |
| --- | --- | --- | --- | --- |
| *TLR1* | NM_001135060.2 | F: 5' CCTCAAAGCAGGGAACAA 3' | 128 | 93.18% |
|  |  | R: 5' GGGCAGATCCAGGTAGATACA 3' |  |  |
| *TLR2* | NM_001048231.1 | F: 5' CTCTGCTACGACGCCTTTG 3' | 109 | 94.51% |
|  |  | R: 5' CAGCTCATACTTGCACCACT 3' |  |  |
| *TLR3* | NM_001135928.1 | F: 5' ATCTGTCCCTGAGCAGCAACC 3' | 153 | 94.70% |
|  |  | R: 5' TCCAGCGTGGTGAGATTCGTC 3' |  |  |
| *TLR4* | NM_001135930.1 | F: 5' GGTTTCAGGAATGCCACTT 3' | 142 | 98.14% |
|  |  | R: 5' CTTTCACCTCTGCCATACTTT 3' |  |  |
| *TLR5* | NM_001135926.1 | F: 5' CTTCCGAAACCTGCCCAATC 3' | 139 | 90.33% |
|  |  | R: 5' TCCAAGCGAGTCAAAGATGC 3' |  |  |
| *Claudin-1* | NM_001185016.1 | F: 5' AATACATTGAGGTCACCGAGTA 3' | 191 | 98.93% |
|  |  | R: 5' GATTAGGCAAGGAAAGGCAC 3' |  |  |
| *Claudin-4* | NM_001185017.1 | F: 5' GCCTTCATCGGCAGCAACAT 3' | 115 | 92.75% |
|  |  | R: 5' CCAGCAGCGAGTCGTACACCTT 3' |  |  |
| *Occludin* | XM_012145891.2 | F: 5' AGTGGTAACTTGGAGACGCTTTC 3' | 107 | 95.17% |
|  |  | R: 5' CCTCCCGTCGTGTAGTCTGTT 3' |  |  |
| *β-Actin* | NM_001009784.2 | F: 5' TCCGTGACATCAAGGAGAAGC 3' | 266 | 91.82% |
|  |  | R: 5' CCGTGTTGGCGTAGAGGT 3' |  |  |
